# Two-pore channel-2 and inositol trisphosphate receptors coordinate Ca^2+^ signals between lysosomes and the endoplasmic reticulum

**DOI:** 10.1016/j.celrep.2023.113628

**Published:** 2023-12-30

**Authors:** Yu Yuan, Vikas Arige, Ryo Saito, Qianru Mu, Gabriela C. Brailoiu, Gustavo J.S. Pereira, Stephen R. Bolsover, Marco Keller, Franz Bracher, Christian Grimm, Eugen Brailoiu, Jonathan S. Marchant, David I. Yule, Sandip Patel

**Affiliations:** 1 Department of Cell and Developmental Biology, University College London, Gower Street, WC1E 6BT London, UK; 2 Department of Pharmacology and Physiology, University of Rochester, 601 Elmwood Avenue, Rochester, NY 14642, USA; 3 Department of Dermatology, Graduate School of Biomedical and Health Sciences, Hiroshima University, Hiroshima 734-8553, Japan; 4 Department of Pharmaceutical Sciences, Jefferson College of Pharmacy, Thomas Jefferson University, 901 Walnut Street, Philadelphia, PA 19107, USA; 5 Department of Pharmacology, Federal University of São Paulo (UNIFESP), São Paulo 04044-020, Brazil; 6 Department of Pharmacy—Center for Drug Research, Ludwig-Maximilian University, Butenandtstrasse 5-13, 81377 Munich, Germany; 7 Walther Straub Institute of Pharmacology and Toxicology, Faculty of Medicine, Ludwig-Maximilian University, Nussbaumstrasse 26, 80336 Munich, Germany; 8 Immunology, Infection and Pandemic Research IIP, Fraunhofer Institute for Translational Medicine and Pharmacology ITMP, 60596 Frankfurt, Germany; 9 Department of Neural Sciences and Center for Substance Abuse Research, Lewis Katz School of Medicine, Temple University, Philadelphia, PA 19140, USA; 10 Department of Cell Biology, Neurobiology & Anatomy, Medical College of Wisconsin, 8701 Watertown Plank Road, Milwaukee, WI 53226, USA; 11 These authors contributed equally; 12 Lead contact

## Abstract

Lysosomes and the endoplasmic reticulum (ER) are Ca^2+^ stores mobilized by the second messengers NAADP and IP_3_, respectively. Here, we establish Ca^2+^ signals between the two sources as fundamental building blocks that couple local release to global changes in Ca^2+^. Cell-wide Ca^2+^ signals evoked by activation of endogenous NAADP-sensitive channels on lysosomes comprise both local and global components and exhibit a major dependence on ER Ca^2+^ despite their lysosomal origin. Knockout of ER IP_3_ receptor channels delays these signals, whereas expression of lysosomal TPC2 channels accelerates them. High-resolution Ca^2+^ imaging reveals elementary events upon TPC2 opening and signals coupled to IP_3_ receptors. Biasing TPC2 activation to a Ca^2+^-permeable state sensitizes local Ca^2+^ signals to IP_3_. This increases the potency of a physiological agonist to evoke global Ca^2+^ signals and activate a downstream target. Our data provide a conceptual framework to understand how Ca^2+^ release from physically separated stores is coordinated.

## In brief

Yuan et al. investigate organelle crosstalk in the genesis of cytosolic Ca^2+^ signals. They resolve a continuum of Ca^2+^ transients from local elementary events driven by lysosomal TPC2 channels, through Ca^2+^-coupled events driven by IP_3_ receptors on the neighboring endoplasmic reticulum, to functionally relevant global signals during physiological cell stimulation.

## Graphical abstract

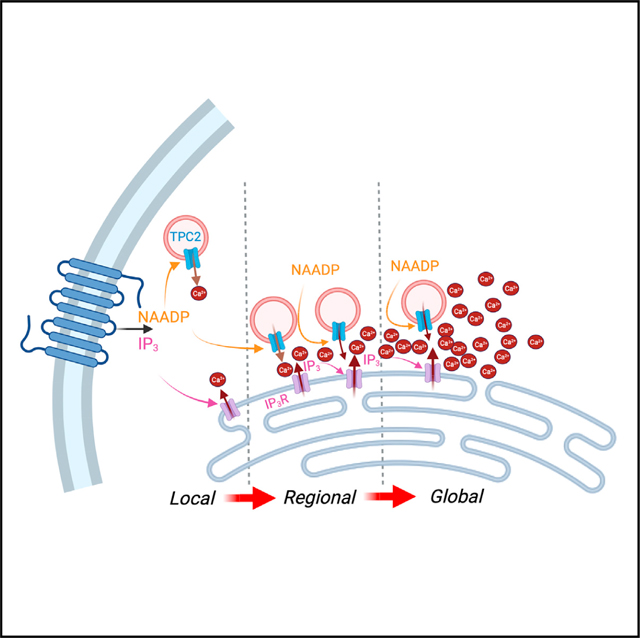

## INTRODUCTION

Changes in cytosolic Ca^2+^ underpin a conserved signaling mechanism that controls numerous cellular events from contraction to gene expression.^1^ Its exquisite specificity, perhaps best exemplified by activation of diametrically opposed Ca^2+^-dependent outcomes in the same cell,^2^ is mediated by complexities in the Ca^2+^ signal. This complexity is manifested both spatially and temporally and is decoded by cellular Ca^2+^ sensors, ensuring fidelity of outputs.^3^ Understanding the genesis of these Ca^2+^ signals is thus key to understanding Ca^2+^-dependent function and, in turn, Ca^2+^-dependent dysfunction in disease.

Ca^2+^ release from intracellular Ca^2+^ stores is initiated by numerous extracellular stimuli such as hormones, neurotransmitters, and growth factors, which are coupled to second-messenger synthesis.^1,4^ As such, these stores represent an important source of physiologically relevant Ca^2+^ signals, particularly in non-excitable cells. The best-characterized pathway is that mediated by IP_3_ produced by receptor-evoked activation of phospholipase C.^4^ IP_3_ targets a family of Ca^2+^ channels, the IP_3_ receptors present on the endoplasmic reticulum (ER). Importantly, the dynamics of Ca^2+^ release are not only regulated by IP_3_ binding, but also by other regulatory inputs including, most importantly, Ca^2+^, which is considered an obligate co-agonist.^5,6^ Elevated cytosolic Ca^2+^ initially enhances channel activity, while at higher Ca^2+^ levels, the channel activity is reduced.^7^ This dynamic interplay between co-agonists, at any given point in time, is thought to underlie the continuum of Ca^2+^ signaling events observed experimentally. For example, at threshold stimulation, ‘‘Ca^2+^ blips’’—elemental events from a few channels—are evoked.^8^ As stimulation intensity increases, localized Ca^2+^ puffs from small clusters of channels are generated as an elevated local Ca^2+^ concentration engages neighboring channels. Summation of activity between close IP_3_ receptor clusters can ultimately lead to global propagating waves.^9^ But Ca^2+^ from other Ca^2+^-permeable channels in the immediate locale can also influence IP_3_ receptor activity. Examples include ryanodine receptors as well as plasma membrane Ca^2+^ channels such as Orai and TRPC.^10,11^

NAADP is a distinct second messenger often co-produced with IP_3_ during signaling.^12,13^ But in contrast to IP_3_, NAADP targets two-pore channels (TPCs) on acidic organelles, such as lysosomes, in many cells.^14–16^ TPCs are highly unusual in dynamically toggling between a Ca^2+^-permeable state upon activation with NAADP to a largely Na^+^-selective state upon activation with the lysosomal-enriched lipid PI(3,5)P_2_.^17–19^ NAADP-evoked Ca^2+^ signals are mediated indirectly through associated NAADP receptors^20–22^ and thought to act locally to regulate vesicular and non-vesicular membrane traffic and organelle morphology.^23^ But these signals are also long thought to be amplified by ER Ca^2+^ channels,^24^ resulting in global Ca^2+^ signals through an ill-defined mechanism.^25,26^ Indeed, in contrast to the ER, we know little of the nature of the putative elementary events stemming from the lysosome^27^ or how they interface with IP_3_-receptor-mediated Ca^2+^ signals. Nevertheless, despite a lack of mechanistic understanding of these events, such coupling is physiologically relevant. In endothelial cells, for example, where the Ca^2+^-mobilizing effects of NAADP have been well characterized, stimuli, such as histamine and vascular endothelial growth factor (VEGF), regulate vascular contractility and angiogenesis by driving nitric oxide production, hyperpolarization, and von Willebrand factor secretion in an NAADP-dependent manner.^28–31^

Here, we addressed interplay between Ca^2+^-release channels resident in lysosomes and the ER, leveraging recently described cell-permeable activators of TPC2,^17^ to study lysosome-derived Ca^2+^ signals in an endogenous setting. Our measurements mechanistically dissect these signals and define a continuum from elementary events associated with TPC2 opening, through sensitization of IP_3_-receptor-driven events on the neighboring ER, to global, cell-wide Ca^2+^ signals evoked by physiological Ca^2+^-mobilizing stimuli.

## RESULTS

### Activation of lysosomal TPC2 evokes local and global Ca^2+^ signals involving the ER

We recently resolved Ca^2+^ signals evoked by activation of endogenous TPC2 channels using synthetic lipophilic cell-permeable TPC2 agonists, TPC2-A1-N and TPC2-A1-P.^17^ These agonists mimic NAADP and PI(3,5)P_2_ action, respectively, and evoke robust channel activation when combined.^18^ Here, we explored organelle crosstalk in the genesis and propagation of these signals.

**Figure 1.**
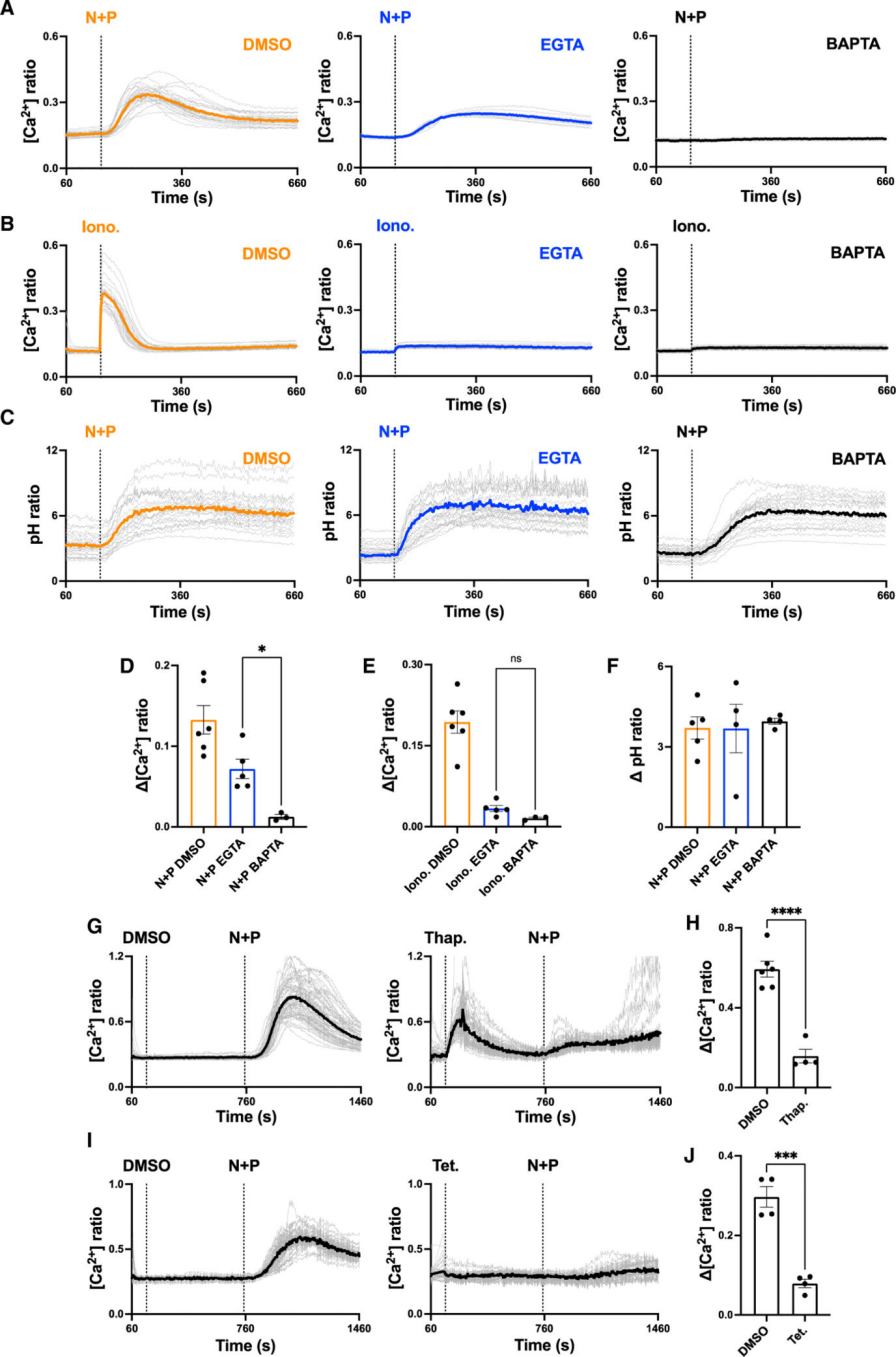
Activation of lysosomal TPC2 evokes local and global Ca^2+^ signals involving the ER (A and B) Effect of DMSO (0.5% [v/v]), EGTA-AM (50 μM), or BAPTA-AM (10 μM) pretreatment on cytosolic Ca^2+^ responses to a combination of TPC2-A1-N (30 μM) and TPC2-A1-P (60 μM) (N + P) (A) or to ionomycin (Iono.; 2 μM) (B) in individual HeLa cells loaded with Fura-2. Each trace is the fluorescence ratio response of a single cell imaged from a typical field of view. The thicker trace is the average of the population. External Ca^2+^ was removed (0 Ca) prior to stimulation. (C) Effect of DMSO (0.5% [v/v]), EGTA-AM (50 μM), or BAPTA-AM (10 μM) pretreatment on lysosomal pH responses to TPC2-A1-N (30 μM) and TPC2-A1-P (60 μM) (N + P) in individual HeLa cells loaded with fluorescein dextran. External Ca^2+^ was removed (0 Ca) prior to stimulation. Each trace is the fluorescence ratio response of a single cell imaged from a typical field of view. The thicker trace is the average of the population. External Ca^2+^ was removed (0 Ca) prior to stimulation. (D‒F) Pooled data (mean ± SEM from 3–6 biological replicates) quantifying the effect of Ca^2+^ chelators on the peak change in cytosolic Ca^2+^ (D and E) and lysosomal pH (F) in response to TPC2 agonists or Iono. Each point represents the mean response of all cells from an independent experiment. *p < 0.05, n.s., not significant (unpaired t test, two-tailed). (G and I) Effect of DMSO (0.1% [v/v]), thapsigargin (1 μM) (G), or tetrandrine (30 μM) (I) on Ca^2+^ signals evoked by a combination of TPC2-A1-N and TPC2-A1-P (N + P) in individual HeLa cells loaded with Fura-2. The agonist concentrations were 30 μM TPC2-A1-N/60 μM TPC2-A1-P and 10 μM TPC2-A1-N/30 μM TPC2-A1-P, respectively. Each trace is the fluorescence ratio response of a single cell imaged from a typical field of view. The thicker trace is the average of the population. External Ca^2+^ was removed (0 Ca) prior to stimulation. External Ca^2+^ was removed (0 Ca) prior to addition to drug treatment. (H and J) Pooled data (mean ± SEM from 4–6 biological replicates) quantifying the effect of thapsigargin (Thap.) (H) and tetrandrine (Tet.) (J) on the peak change in cytosolic Ca^2+^ in response to TPC2 agonists. Each point represents the mean response of all cells from an independent experiment. ***p < 0.001, ****p < 0.0001 (unpaired t test, two-tailed).

Consistent with our previous analysis,^18^ stimulation of HeLa cells with a combination of TPC2 agonists evoked a robust Ca^2+^ signal that was readily detected by epifluorescence microscopy of the Ca^2+^ indicator Fura-2 (Figure 1A). To dissect the origins of these Ca^2+^ signals, we first examined the effects of the Ca^2+^ chelator EGTA. In initial experiments, we titrated the concentration of its acetoxymethyl ester (AM) to define conditions able to block Ca^2+^ signals in response to the Ca^2+^ ionophore, ionomycin (Figure 1B). Under these conditions, the response to the agonist combination was substantially reduced but not eliminated (Figure 1A), with a readily resolvable residual signal.

Because EGTA is a slow chelator that is considered ineffective in buffering Ca^2+^ around the open pore of channels,^32^ we considered that the residual signal was a local signal. To test this, we examined the effect of the fast Ca^2+^ chelator, BAPTA. As shown in Figure 1A, pretreating cells with 10 μM BAPTA-AM fully blocked the TPC2-evoked response as well that to ionomycin (Figure 1B).

To test if the blocking effects of the chelators was due to a direct action on TPC2, we inferred TPC2 activity by measuring luminal pH, which increases upon TPC2 activation in response to TPC2-A1-N.^17^ As shown in Figure 1C, TPC2 activation evoked a time-dependent increase in luminal pH in cells loaded with fluorescein dextran. This response was not inhibited by EGTA or BAPTA. Individual wavelengths used to derive ratiometric images are shown in Figure S1. Thus, TPC2 channel activation is intact upon chelator treatment. The differential effects of the Ca^2+^ chelators on cytosolic Ca^2+^ and lysosomal pH are quantified in Figures 1D–1F.

To probe the involvement of the ER in Ca^2+^ signals evoked by TPC2 activation, we depleted ER Ca^2+^ stores using the SERCA inhibitor thapsigargin. As shown in Figures 1G and 1H, stimulation of HeLa cells with thapsigargin (1 μM) in the absence of external Ca^2+^ evoked a transient Ca^2+^ signal consistent with leak of stored Ca^2+^. Subsequent stimulation with the TPC2 agonist combination evoked a substantially smaller response than in cells treated with vehicle. Therefore, the majority of signal evoked by TPC2 agonist involves Ca^2+^ release from the ER.

We previously showed that Ca^2+^ signals in response to the TPC2 agonists were TPC2 dependent based on the use of inactive analogs, dominant-negative TPC2 and TPC2 knockout cells generated using CRISPR-Cas9.^18^ To provide further evidence that the Ca^2+^ response was TPC2 derived, we examined the effect of the TPC2 blocker tetrandrine.^33^ Tetrandrine, like thapsigargin, also blocked the TPC2-agonist-evoked Ca^2+^ signal (Figures 1I and 1J). This block together with demonstrable engagement of lysosomes (Figures 1C, 1F, and S1) confirm on-target effects of the TPC2 agonists.

In sum, these data resolve both local and global components of the Ca^2+^ signal evoked upon TPC2 activation and uncover a significant role for the ER in lysosomal-initiated Ca^2+^ signaling.

### ER and lysosomal channel levels set the timing of TPC2-evoked Ca^2+^ signals

**Figure 2.**
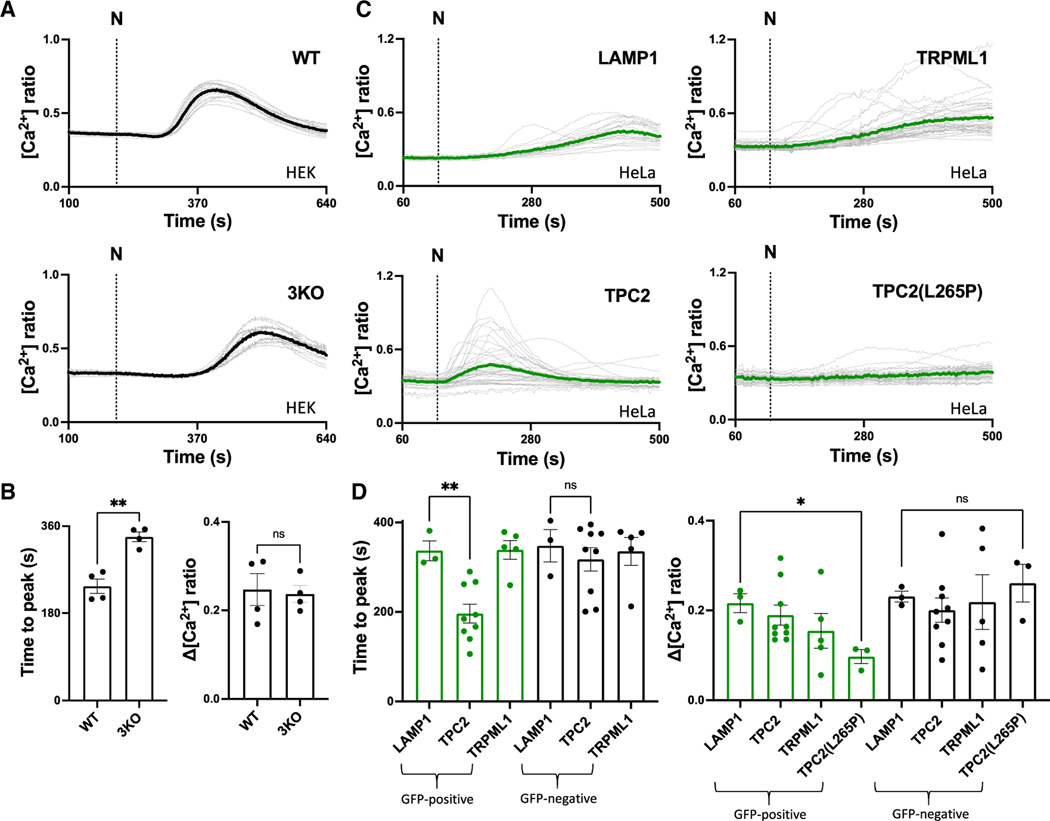
ER and lysosomal channel levels set the timing of TPC2-evoked Ca^2+^ signals (A) Effect of TPC2-A1-N (15 μM) on cytosolic Ca^2+^ in individual wild-type (WT) and IP_3_ receptor knockout (3KO) HEK-293 cells loaded with Fura-2. Each trace is the fluorescence ratio response of a single cell imaged from a typical field of view. The thicker trace is the average of the population. External Ca^2+^ was removed prior to stimulation. (B) Pooled data (mean ± SEM from 4 biological replicates) quantifying the time to peak and the maximal change of the cytosolic Ca^2+^ signals in response to TPC2-A1-N in HEK-293 cells. Each point represents the mean response of all cells from an independent experiment. **p < 0.01, n.s., not significant (unpaired t test, two-tailed). (C) Effect of TPC2-A1-N (30 μM) on cytosolic Ca^2+^ in individual HeLa cells transiently transfected with LAMP1-GFP, TPC2-GFP, TRPML1-GFP, or TPC2^L265P^-GFP and loaded with Fura-2. Each trace is the fluorescence ratio response of a single cell imaged from a typical field of view. External Ca^2+^ was removed (0 Ca) prior to stimulation. (D) Pooled data (mean ± SEM from 3–9 biological replicates) quantifying the maximal change and the time to peak of the cytosolic Ca^2+^ signals in response to TPC2-A1-N in HeLa cells. Data are segregated into cells that were GFP positive and -negative. Each point represents the mean response of all cells from an independent experiment. *p < 0.05, **p < 0.01, n.s., not significant (one-way ANOVA followed by Dunnett’s test or Kruskal-Wallis test followed by Dunn’s test).

To explore mechanisms underpinning lysosome-ER crosstalk, we focused on the role of IP_3_ receptors, which are ubiquitous ER Ca^2+^-release channels.^34,35^ For these experiments, we used HEK cells where all three IP_3_ receptor subtypes were knocked out by CRISPR-Cas9.^36^ Similar to HeLa cells,^18^ stimulation of wild-type HEK cells with TPC2-A1-N evoked low-amplitude Ca^2+^ signals (Figure 2A). TPC2-A1-N also evoked Ca^2+^ signals in IP_3_ receptor knockout cells (Figure 2A). But these signals were substantially delayed in terms of time to reach their peak, although their amplitude was not affected (Figures 2A and 2B). Similar results were obtained with the agonist combination (Figures S2A and S2B). Thus, IP_3_ receptors appear to set the timing of the Ca^2+^ signals.

In a converse set of experiments, we examined the effect of overexpressing TPC2 versus overexpressing the non-channel lysosomal protein LAMP1 as a negative control. As shown in Figure 2C, cells expressing TPC2 responded to TPC2-A1-N much sooner compared to cells expressing LAMP1. There was no significant difference in the amplitude of the signals, although the responses in individual TPC2-expressing cells were variable, probably due to differing expression levels resulting from transient transfection (Figures 2C and 2D). More prompt responses were also obtained with the agonist combination upon TPC2 overexpression (Figures S2C and S2D). Thus, promoting Ca^2+^ release through TPC2 overexpression selectively affects the timing of the Ca^2+^ signal. This effect was specific to TPC2, as overexpressing the unrelated lysosomal Ca^2+^ channel TRPML1 had no effect on the timing of Ca^2+^ release or signal amplitude compared to LAMP1 overexpression (Figures 2C and 2D). We also examined the effect of overexpressing TPC2 mutated in the pore.^37^ TPC2^L265P^ inhibited TPC2-A1-N-evoked Ca^2+^ signals (Figures 2C and 2D), consistent with dominant-negative activity and further confirming a specific effect of TPC2-A1-N on endogenous TPC2. Responses in neighboring untransfected cells in the populations were not different (Figures 2D and S3).

Taken together, these experiments show that both IP_3_ receptors and TPC2 modulate the timing of lysosomal-derived-Ca^2+^ signals.

### IP_3_ receptors locally couple to TPC2

To further explore mechanisms underpinning lysosome-ER crosstalk, we performed high-resolution total internal reflection fluorescence (TIRF) microscopy of cells loaded with Cal520 to resolve elementary Ca^2+^ signals. Measurements were made during the first 30 s of stimulation, where Ca^2+^ signals were not readily detectable by epifluorescence microscopy.

**Figure 3.**
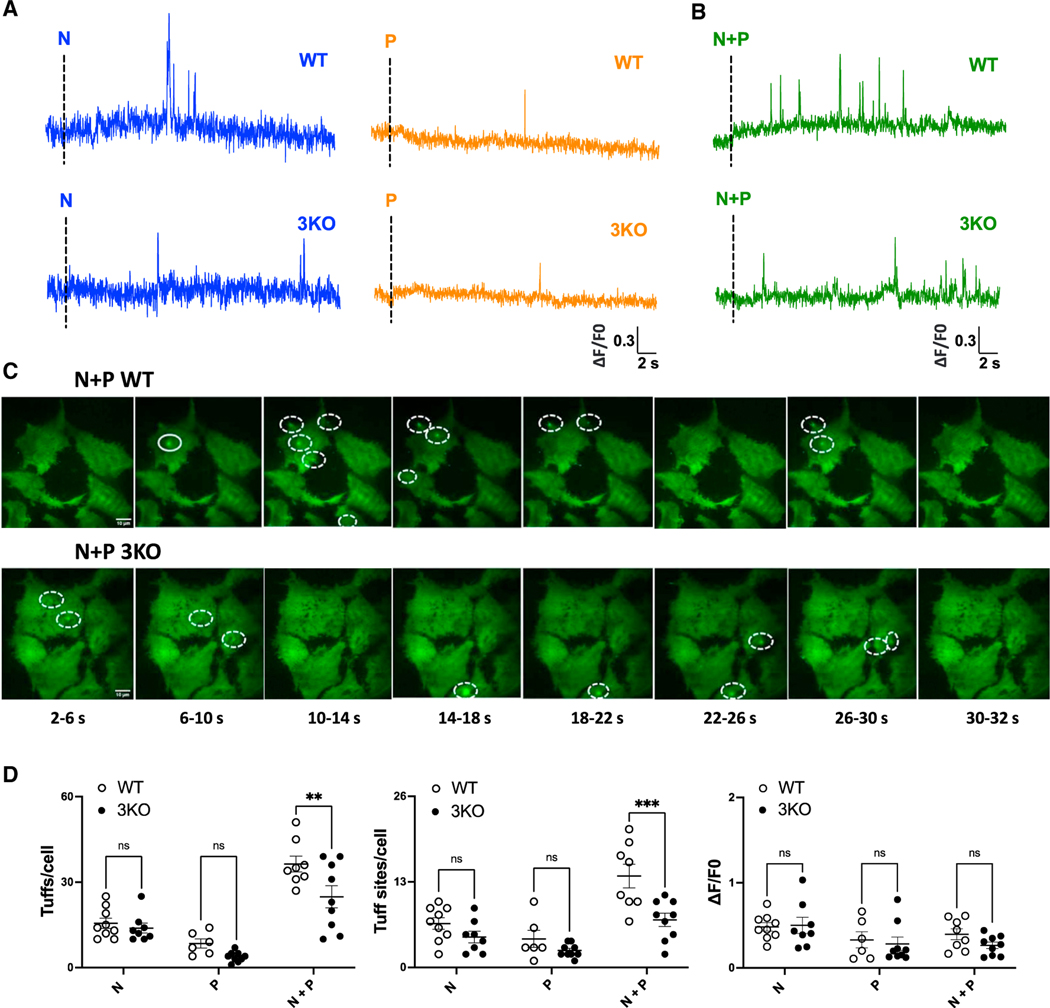
IP_3_ receptors locally couple to TPC2 (A and B) Effect of TPC2-A1-N (N, 30 μM), TPC2-A1-P (P, 30 μM) (A), or a combination of the two (B) on subcellular Ca^2+^ levels of individual WT and 3KO HEK-293 cells loaded with Cal520 and EGTA-AM. Representative time courses of fluorescence changes from the center ofsingle tuff sites (1 × 1 μm) in response to indicated reagents. (C) Maximal intensity projections of Cal520 fluorescence at 4 s intervals after stimulation of WT and 3KO with the agonist combination. Elementary Ca^2+^ signals are highlighted. (D) Pooled data (mean ± SEM from 6–9 biological replicates) quantifying the number of tuff events, sites detected per cell, and the amplitude of the events in response to the indicated stimulus. Each point represents data from an individual cell. **p < 0.01, ***p < 0.001, n.s., not significant (two-way ANOVA followed by Bonferroni’s test).

Consistent with our previous analysis,^18^ stimulation of HEK cells with TPC2-A1-N or TPC2-A1-P evoked transient, spatially resolved Ca^2+^ signals (Figure 3A). These ‘‘tuffs’’ were more prevalent upon activation of TPC2 with TPC2-A1-N than TPC2-A1-P, consistent with biasing of the channel by TPC2-A1-N to a Ca^2+^-permeable state.^17^ To determine whether these tuffs represent pure TPC2-evoked responses or secondary signals through IP_3_ receptors (or both), we examined the effect of IP_3_ receptor knockout. As shown in Figure 3A, tuffs were readily observable in the knockout cells. Thus, tuffs likely represent elementary TPC2-dependent Ca^2+^ signals.

We next tested the requirement of IP_3_ receptors in response to TPC2 co-activation, which goes on to generate global Ca^2+^ signals (Figure 1). In wild-type HEK cells, a combination of TPC2-A1-N and TPC2-A1-P significantly increased activity (Figures 3B and 3C; Video S1), as reported previously.^18^ In contrast, the TPC2 agonist combination had more modest effects in the IP_3_ receptor knockout cells (Figures 3B and 3C; Video S2).

This analysis is summarized in Figure 3D. The data show that the number of tuffs recorded and the number of tuff sites/cell, when cells were stimulated with TPC2-A1-N, were not affected by IP_3_ receptor knockout (Figure 3D). Both parameters were increased in response to the agonist combination in wild-type cells but less so in IP_3_ receptor knockout cells. Thus, both IP_3_-receptor-dependent events and IP_3_-receptor-independent events occur during TPC2 activation. Neither the amplitude (Figure 3D) nor the kinetics (Figure S4A) of events was affected.

In summary, we provide genetic evidence that lysosome-derived Ca^2+^ signals are locally amplified by the ER.

### TPC2 sensitizes IP_3_ receptors in an agonist-selective manner

**Figure 4.**
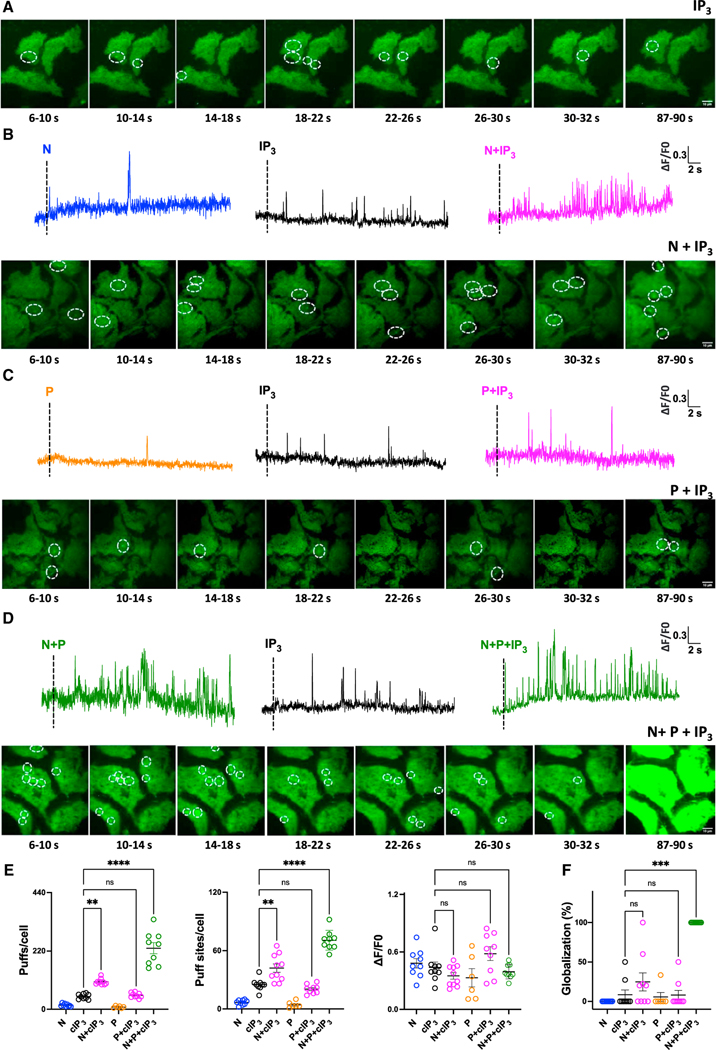
TPC2 sensitizes IP_3_ receptors in an agonist-selective manner (A) Effect of UV photolysis of caged-iIP_3_ (IP_3_) (0.5 μM) on subcellular Ca^2+^ levels of individual HEK-293 cells loaded with Cal520. Maximal intensity projections of Cal520 fluorescence at 4 s intervals (200 frames) after stimulation are shown. Elementary Ca^2+^ signals are highlighted. (B‒D) Effect of UV photolysis of caged-IP_3_ (IP_3_) (0.5 μM) in the absence or presence of TPC2-A1-N (N, 30 μM, B), TPC2-A1-P (P, 30 μM, C), or a combination of the two (D) on subcellular Ca^2+^ levels of individual HEK-293 cells loaded with Cal520. Representative time courses of fluorescence changes from the center of single tuff sites (1 × 1 μm) in response to indicated reagents. Maximal intensity projections of Cal520 fluorescence at 4 s intervals after stimulation are shown below. Elementary Ca^2+^ signals are highlighted. (E and F) Pooled data (mean ± SEM from 6–10 biological replicates) quantifying the number of tuff events, sites detected per cell, the amplitude of the events (E), and the proportion of cells in which Ca^2+^ signals globalized within 90 s (F) in response to the indicated stimulus. Each point represents data from an individual cell. **p < 0.01, ***p < 0.001, ****p < 0.0001, n.s., not significant (one-way ANOVA followed by Dunnett’s test or Kruskal-Wallis test followed by Dunn’s test).

In a converse approach, we examined the effect of TPC2 activation on IP_3_-evoked Ca^2+^ responses. To do this, we loaded cells with a submaximal concentration of ciIP_3_,a caged form of IP_3_, and liberated the active compound by UV photolysis. As shown in Figure 4A, activation of IP_3_ receptors in this way evoked elementary events (puffs; Video S3) that were similar to those evoked by activation of TPC2 (tuffs). When ciIP_3_ was uncaged in the presence of TPC2-A1-N, there was a substantial increase in activity (Figure 4B; Video S4). Thus, TPC2-A1-N synergizes with IP_3_ to regulate local Ca^2+^ signals.

We performed similar experiments with TPC2-A1-P. In marked contrast to TPC2-A1-N, TPC2-A1-P had no effect on the response to uncaging ciIP_3_ (Figure 4C; Video S5). This was despite a clear synergism between TPC2-A1-P and TPC2-A1-N recorded under identical conditions (Figures 3A and 3B). Thus, crosstalk with IP_3_ receptors and TPC2 is only apparent with TPC2-A1-N (which renders TPC2 Ca^2+^ permeable) and not with TPC2-A1-P (which renders TPC2 largely Na^+^-selective).

We also analyzed the effectof IP_3_ together with the TPC2 agonist combination. As shown in Figure 4D, there was a further increase in activity relative to IP_3_ combined with TPC2-A1-N. Notably, the triple combination evoked global Ca^2+^ signals at extended time points (Figure 4D; Video S6), which was not observed when IP_3_ was increased in the presence of TPC2-A1-N and TPC2-A1-P.

The effects of various channel agonist combinations on cytosolic Ca^2+^ are quantified in Figures 4E, 4F, and S4B. This analysis showed that the increased subcellular activity by IP_3_ in the presence of TPC2-A1-N results from an increase in the both the number of events and sites per cell, but not amplitude (Figure 4E), with a tendency for the signals to globalize (Figure 4F). This activity is further increased by TPC2-A1-P, which invariably results in cell-wide Ca^2+^ signals. The kinetics of events are similar (Figure S4B).

In summary, we reveal that IP_3_ receptors can be modulated by TPC2 in an agonist-selective manner to control both local and global Ca^2+^ signals.

### Lysosome-ER crosstalk regulates physiological Ca^2+^ signals

To probe the physiological relevance of lysosome-ER crosstalk, we examined the effect of TPC2 activation on Ca^2+^ signals evoked by an IP_3_-forming agonist.

**Figure 5.**
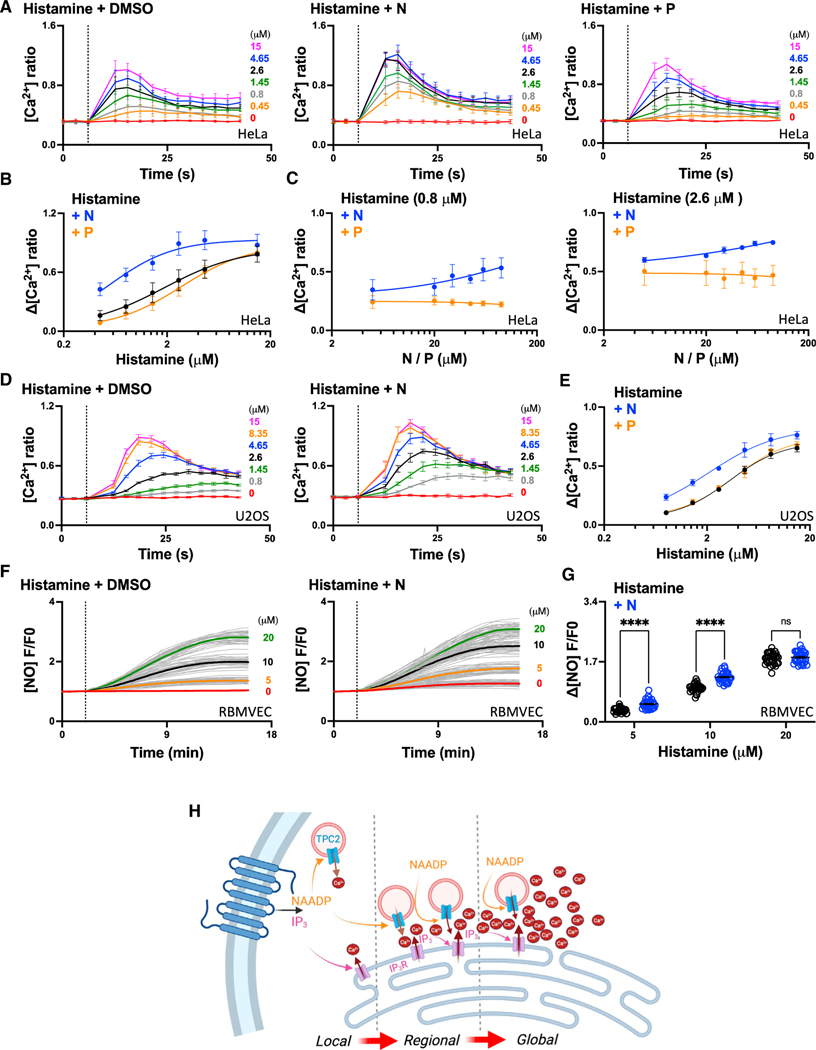
Lysosome-ER crosstalk regulates physiological Ca^2+^ signals (A‒C) Effect of TPC2-A1-N or TPC2-A1-P on histamine-induced Ca^2+^ signals in HeLa cells loaded with Fura-2. (A) Time course showing the effect of co-stimulating cell populations in an automated manner with increasing concentrations of histamine and a fixed concentration of DMSO (0.1% [v/v]), TPC2-A1-N (N, 30 μM), or TPC2-A1-P (P, 60 μM). (B) Concentration-effect relationship for peak histamine responses in the presence of indicated reagents. (C) Effect of co-stimulating cells with increasing concentrations of TPC2-A1-N or TPC2-A1-P at a fixed concentration of histamine (0.8 μM, left, or 2.6 μM, right). Data are expressed as mean ± SEM from 3–4 biological replicates. (D and E) Effect of TPC2-A1-N or TPC2-A1-P on histamine-induced Ca^2+^ signals in U2OS cells loaded with Fura-2. (D) Time course showing the effect of co-stimulating cell populations in an automated manner with increasing concentrations of histamine and a fixed concentration of DMSO (0.1% [v/v]) or TPC2-A1-N (10 μM). (E) Concentration-effect relationship for peak histamine responses in the presence of TPC2-A1-N (10 μM) or TPC2-A1-P (20 μM). Data are expressed as mean ± SEM from 3–4 biological replicates. (F and G) Effect of TPC2-A1-N on histamine-induced NO production in rat brain microvascular endothelial cells (RBMVECs) loaded with DAF 2A. (F) Time course data showing the effect of co-stimulating cells with increasing concentrations of histamine and a fixed concentration of DMSO (0.1% [v/v]) or TPC2-A1-N (N, 10 μM). Each trace is the fluorescence response of a single cell. The thicker trace is the average of the population. (G) Concentration-effect relationship for peak histamine responses in the presence of indicated reagents. Data were corrected for basal NO production in the absence of histamine. Data are expressed as mean ± SEM from 27–33 cells. ****p < 0.0001, n.s., not significant, two-way ANOVA followed by Bonferroni’s test. (H) Proposed model whereby local Ca^2+^ signals stemming from the lysosome via TPC2 and the ER via IP_3_ receptors form regional intermediaries that precede global Ca^2+^ signals upon cellular stimulation. Created with BioRender.com.

HeLa cells were stimulated with histamine, which couples to Gq and phospholipase C. Responses were measured using Fura-2 and automated plate-reading equipped with microfluidics. The effect of a range of histamine concentrations is shown in Figure 5A. The responses to histamine peaked within the 40 s recording period. Over this time frame, TPC2-A1-N had no detectable effect on cytosolic Ca^2+^ concentration. But when cells were stimulated with histamine in the presence of TPC2-A1-N, the response to histamine was exaggerated. Thus, TPC2-A1-N synergizes with endogenously produced IP_3_ to regulate global Ca^2+^ signals.

The effect of TPC2-A1-P on histamine responses was examined in parallel (Figure 5A). Histamine responses were largely unaffected by co-stimulation. The marked specificity between the agonists mirror results with exogenous IP_3_ (Figure 4).

Full concentration-effect relationships for histamine-evoked Ca^2+^ signals in the absence and presence of either TPC2-A1-N or TPC2-A1-P are shown in Figure 5B. This analysis revealed an approximate 5-fold decrease in EC_50_, from ~2 μM in the presence of vehicle to ~0.4 μM in the presence of TPC2-A1-N, and a modest (<2-fold) increase in the presence of TPC2-A1-P.

To further characterize this sensitization, we examined the effect of increasing concentrations of TPC2-A1-N or TPC2-A1-P on a submaximal concentration of histamine. Figure 5C shows the response to a low concentration (0.8 μM) of histamine. TPC2-A1-N increased the response in a concentration-dependent manner, whereas TPC2-A1-P modestly decreased it. Essentially similar results were obtained when the histamine concentration was increased (to 2.6 μM) (Figure 5C).

We also examined the effects of TPC2 activation on histamine responses in U2OS cells, which mount robust responses to TPC2-A1-N.^38^ Here, we used a relatively low concentration of TPC2-A1-N (10 μM) such that it had no detectable effect on cytosolic Ca^2+^ concentration over the time frame of histamine stimulation. As in HeLa, cells TPC2-A1-N increased the responses to histamine in U2OS cells (Figure 5D). Again, this was due to a reduction in the EC_50_ for histamine (2- to 4-fold) and was selective for TPC2-A1-N over TPC2-A1-P (Figure 5E).

We validated the above findings at the single-cell level (Figure S5). A submaximal concentration of histamine evoked a prompt Ca^2+^ signal in HeLa (Figure S5A) and U2OS (Figure S5B) cells. Co-stimulation with TPC2-A1-N increased the peak response ~2-fold in both cell types (Figure S5), thereby mirroring results obtained with the automated assay.

Finally, we examined the consequence of the sensitized Ca^2+^ signals evoked by TPC2-A1-N. For these experiments, we used primary endothelial cells and measured NO production in response to histamine using DAF-FM. NO is produced by endothelial nitric oxide synthase in a Ca^2+^-dependent manner. As shown in Figure 5F, histamine induced a concentration-dependent increase in NO production. When cells were co-stimulated with TPC2-A1-N, the NO responses were enhanced at low, but not high, histamine concentrations (Figures 5F and 5G). This is consistent with a left-ward shift in the concentration-effect relationships for NO production by histamine.

In summary, these data show that activating local Ca^2+^ fluxes by lysosomal TPC2 sensitizes global Ca^2+^ signals and a downstream response evoked by ER IP_3_ receptors upon physiological stimulation.

## DISCUSSION

Overall, this work defines a continuum of Ca^2+^ signals from local elementary events derived from lysosomal TPC2, through Ca^2+^-coupled events requiring IP_3_ receptors on the neighboring ER, to global functionally relevant signals during physiological cell stimulation (Figure 5H).

NAADP-evoked Ca^2+^ signals are thought to comprise a primary event driven by NAADP-regulated channels and a secondary event as ER Ca^2+^ channels are engaged. This is based on early evidence showing blockade of NAADP-mediated Ca^2+^ signals by blockers of ER Ca^2+^ channels.^39^ But rarely have the putative triggering events been directly measured. In pancreatic acinar cells, use of Ca^2+^-activated Cl^—^ currents as a sensitive measure of local Ca^2+^ spiking in the secretory pole could not resolve any NAADP-evoked activity in the absence of ER functionality.^24^ This, coupled with the reported direct effects of NAADP on ER ryanodine receptors,^40^ leaves gaps in our understanding of how lysosomal and ER Ca^2+^ stores are functionally coupled.

Here, we took full advantage of cell-permeable activators of TPC2 to probe lysosome-ER crosstalk in live cells. TPC2-A1-N is a functional NAADP mimetic inducing almost identical biophysical currents through TPC2^17^ but independently of NAADP-binding proteins.^38^ As shown recently, activation of TPC2 with TPC2-A1-N and its co-ligand induces robust Ca^2+^ permeability.^18^ Our results with Ca^2+^ chelators provide direct evidence that the resulting endogenous TPC2-evoked Ca^2+^ signals evoked in cells comprise a local and a global component (Figure 1). The former has been inferred indirectly, mostly through functional outputs such as endo-lysosomal morphology. This component likely corresponds to lysosomal Ca^2+^ release evoked by stimulation of the channel with TPC2-A1-N. The global component is unlikely to be solely represented by lysosomal Ca^2+^ release given the relatively small volume of the lysosome. In accord, depletion of ER Ca^2+^ stores profoundly inhibited the response despite its lysosomal origin. Importantly, we provide genetic evidence that such coupling between stores involves IP_3_ receptors given the delaying of TPC2-A1-N-evoked Ca^2+^ signals by IP_3_ receptor knockout (Figure 2).

Building on our previous high-resolution analysis, we characterized the local events evoked upon TPC2 activation (Figure 3). We did so in an IP_3_ receptor knockout background, thus providing direct evidence that tuffs are indeed elementary events mediated by TPC2. Although tuffs evoked by TPC2-A1-N alone were IP_3_-receptor independent, this was not the case for tuffs evoked by the TPC2 agonist combination, as there was a significant decrease in tuff frequency and number of tuff sites upon IP_3_ receptor knockout (Figure 3). These data uncover local recruitment of IP_3_ receptors by TPC2, which partially accounts for the demonstrable synergism between TPC2-A1-N and TPC2-A1-P. This is in addition to direct effects of the agonist combination on TPC2 at the channel level, where there is a selective increase in permeability of Ca^2+^ over Na^+^.^18^ Importantly, recruitment of IP_3_ receptors by TPC2 in this activation state occurred without overt increases in IP_3_ production. Coupling therefore likely reflects IP_3_ receptor activity at basal levels of IP_3_.

Further evidence that TPCs and the IP_3_ receptor cooperate comes from experiments where cells were co-stimulated with TPC2-A1-N and IP_3_ (Figure 4). Both the number of elementary events and the number of sites from which they originate were increased. This synergism is remarkably similar to that when cells were co-stimulated with TPC2-A1-N and TPC2-A1-P and is consistent with a previous study showing potentiation of IP_3_ responses by NAADP.^41^ Thus, local Ca^2+^ signals can be tuned at either side of the lysosomal-ER interface. Importantly, sensitization of IP_3_ receptors by TPC2 was evident during physiological stimulation of cells with an IP_3_-forming agonist, with marked left-ward shifts in concentration-effect relationships for histamine-mediated Ca^2+^ and NO signals (Figure 5). Such sufficiency of acidic organelles in regulating IP_3_ receptors, together with the necessity of acidic organelles for IP_3_-forming agonists to mount global responses,^13,42,43^ cements organelle crosstalk during physiological signaling.

IP_3_ and ryanodine receptor activity is biphasically regulated by Ca^2+^ such that low concentrations of Ca^2+^ stimulate activity, whereas higher concentrations inhibit it.^6^ A long-held idea is that NAADP in effect highjacks the positive effect of Ca^2+^ on ER Ca^2+^-release channels to mount global responses.^24^ This would be akin to coupling of voltage-gated Ca^2+^ channels on the plasma membrane with ryanodine receptors on the sarcoplasmic reticulum during cardiac excitation-contraction coupling.^44^ Direct evidence for coupling between lysosomes and the ER in this way, however, is lacking. Our data, leveraging the malleable ion selectivity of TPC2, provide missing evidence for such a Ca^2+^-induced Ca^2+^-release model. TPC2-A1-P is a functional mimetic of PI(3,5)P_2_ at TPC2 that likely binds to an overlapping site.^17,45^ The Ca^2+^ permeability of TPC2 in the presence of TPC2-A1-P measured electrophysiologically is low, and TPC2-A1-P induces infrequent elementary Ca^2+^ events in cells. Importantly, TPC2-A1-P failed to synergize with IP_3_ either directly (through uncaging experiments) or indirectly (through histamine-evoked IP_3_ production). This dichotomy between TPC2-A1-P (‘‘Na^+^ agonist’’) and TPC2-A1-N (‘‘Ca^2+^ agonist’’) points to Ca^2+^ as the link between TPC2 and IP_3_ receptors.

One intriguing aspect of our data relates to the mode by which TPC2 and IP_3_ receptors communicate, pointing to a digital system of signaling. For example, promoting triggering (by increasing TPC2 levels) or demoting amplification (by decreasing IP_3_ receptor levels) did not affect the amplitude of the lysosome-derived signals only their timing (Figure 2). Similarly, synergy between TPC2-A1-N and IP_3_ (and with TPC2-A1-P too) was amplitude independent, instead manifesting as an increase in the number of elementary events recorded (Figure 4). Indeed, kinetically, events mediated by the various cues and their combinations were not readily distinguishable (Figure S4). Further, work is required to understand how these events are decoded. Indeed, it is interesting to note that elementary events evoked by IP_3_ through each of the three IP_3_ receptor subtypes are also not dissimilar.^46^

### Limitations of the study

Cell-permeable TPC2 agonists used here to mimic the effects of NAADP and PI(3,5)P_2_ have been most enabling for probing the mechanisms underpinning lysosomal Ca^2+^ signaling in live cells. But these agonists are mimics, so how faithfully they recapitulate the actions of their natural counterparts merits consideration. Certainly TPC2-A1-P, which synergizes with TPC2-A1-N just like PI(3,5)P_2_ does with NAADP, shares, at least in part, common molecular determinants of action at TPC2 with PI(3,5)P_2_.^17^ This is not the case for TPC2-A1-N and NAADP since NAADP requires NAADP-binding proteins JPT2 and Lsm12, which TPC2-A1-N bypasses.^40^ Thus, the results presented here require validation using natural messengers. Related to this is the specificity of the TPC2 agonists. To mitigate against potential off-target effects, we used both chemical (tetrandrine) and molecular (dominant-negative TPC2) inhibition approaches to validate TPC2-A1-N-evoked Ca^2+^ signals. But corroborating genetic knockout of TPC2 as reported previously^17,18^ is currently lacking in the cell types used here. Finally, we have focused on TPC2 and IP_3_ receptors. But lysosomes and the ER possess a number of other Ca^2+^-permeable channels, namely TRPML1 and ryanodine receptors, which might also be functionally coupled.^47^ Thus, more work is required to understand the full scope of interorganelle crosstalk between acidic and ER Ca^2+^ stores.

To conclude, we provide insight into the molecular and organellar makeup of lysosomal-derived Ca^2+^ signals and establish a hierarchal framework for understanding how cellular Ca^2+^ signals are coordinated.

## STAR★METHODS

### RESOURCE AVAILABILITY

#### Lead contact

Further information and requests for resources and reagents should be directed to and will be fulfilled by the lead contact, Sandip Patel (patel.s@ucl.ac.uk).

#### Materials availability

No newly generated materials are associated with the paper.

#### Data and code availability

- All data reported in this paper will be shared by the lead contact upon request.
- This paper does not report original code.
- Any additional information required to reanalyze the data reported in this paper is available from the lead contact upon request.

### EXPERIMENTAL MODEL AND STUDY PARTICIPANT DETAILS

#### Cells

HeLa cells, U2OS cells, wild-type HEK-293 and HEK-293 cells engineered using CRISPR-Cas technology to lack all the three-native endogenous IP_3_ receptors^36^ were maintained in Dulbecco’s Modified Eagle Medium, supplemented with 10% (v/v) Fetal Bovine Serum, 100 μg/mL streptomycin and 100 units/mL penicillin (all from Invitrogen) at 37°C in a humidified atmosphere with 5% CO_2_. Rat brain microvascular endothelial cells (Cell Applications, Inc. (San Diego, CA, USA) were cultured in rat brain endothelial cell basal medium and rat brain endothelial cell growth supplement in flasks coated with attachment factor according to the manufacturer’s instructions (Cell Applications, Inc.)^51,52^

For single cell epifluorescence imaging, HeLa and U2OS cells were plated onto round 13 mm diameter coverslips (Academy) coated with poly-L-lysine (20 μg/mL, Sigma). HeLa cells were transiently transfected with plasmids 18–26 h prior to imaging, using lipofectamine 2000 (Invitrogen) according to the manufacturer’s instructions. Rat brain microvascular endothelial cells were plated onto round 25 mm diameter coverslips (Warner) coated with human fibronectin (50 μg/mL, Corning). HEK-293 cells were plated onto round 15 mm diameter coverslips (Warner).

For TIRF imaging, HEK-293 cells were plated onto round 15 mm diameter coverslips (Warner) coated with poly-D-lysine (100 μg/mL, Sigma).

For plate reading, cells were plated onto opaque-walled 96 well microplates (Corning).

### METHOD DETAILS

#### Single cell imaging

Cytosolic Ca^2+^ in HeLa and U2OS cells was measured using Fura-2 (from Biotium). Ca^2+^ imaging experiments were performed at room temperature in HEPES-buffered saline (HBS1) containing 10 mM NaHEPES, 1.25 mM KH_2_PO_4_, 2 mM MgSO_4_, 3 mM KCl, 156 mM NaCl, 2 mM CaCl_2_ and 10 mM glucose (pH 7.4; all from Sigma-Aldrich). For dye loading, cells were incubated with Fura-2-AM. (2.5 μM) and 0.005% (v/v) pluronic acid (from Invitrogen) for 1 h in HBS1. In case of Ca^2+^ chelator treatment, after 1 h Fura-2 loading, HeLa cells were washed three times in HBS1 followed by 45-min treatment with EGTA-AM (50 μM; ChemCruz Biotechnology) or BAPTA-AM (10 μM; Cayman) in HBS1 at room temperature. After that, cells were washed in HBS1 and left in HBS1 for another 30 min at room temperature to allow de-esterification of AM esters. Where indicated, majority of the experiments were performed in nominally Ca^2+^-free HBS1 where CaCl_2_ was omitted.

Cytosolic Ca^2+^ in HEK-293 was measured using Fura-2 (from Invitrogen). Ca^2+^ imaging experiments were performed at room temperature in HEPES-buffered saline (HBS2) containing 137 mM NaCl, 0.56 mM MgCl_2_, 4.7 mM KCl, 1 mM Na_2_HPO_4_, 10 mM HEPES, 5.5 mM glucose, and 1.26 mM CaCl_2_ (pH 7.4). Cells were incubated with Fura-2-AM (2 μM) in HBS2 supplemented with 1% BSA for 30 min.

Lysosomal pH in HeLa cells was measured using fluorescein in nominally Ca^2+^-free HBS1 at room temperature. Cells were loaded with Fluorescein-dextran (0.1 mg/mL; MW 10,000; from Invitrogen) by endocytosis overnight in culture followed by up to 10 h chasing period in dextran-free culture medium.

Intracellular nitric oxide (NO) in rat brain microvascular endothelial cells was measured using DAF-FM (Life Technologies Corporation, Eugene, OR). NO imaging experiments were performed at 21°C in Hanks’ Balanced Salt Solution (HBSS) (Corning). For dye loading, cells were incubated with DAF-FM diacetate (0.5 μM) for 45 min in HBSS.^53,54^

For HeLa and U2OS cells, after transfection and/or dye loading, cells were washed in HBS1 and were subsequently mounted in a 1 mL imaging chamber (Biosciences Tools) for microscopy. Epifluorescence images were acquired every 3 s. For some Fura-2 measurements (Figures 1A, 1B, S2C, and S5A) and Fluorescein measurements (Figures 1C, S1A, and S1B), images were captured using a Megapixel monochrome cooled coupled device camera attached to an Olympus IX73 inverted fluorescence microscope fitted with a CoolLED multiple wavelength LED source under the control of MetaFluor 7.10.3.279 software. Fura-2 was excited at 340/380 nm and emitted fluorescence was captured using a 425 nm long-pass filter with a 20X objective. Fluorescein was excited at 470 nm/405 nm and emitted fluorescence was captured using a 510 nm long-pass filter at 20X magnification. For other Fura-2 measurements (Figures 1G, 1I, 2C, S3, and S5B), images were captured with a cooled coupled device camera (TILL photonics) attached to an Olympus IX71 inverted fluorescence microscope fitted with a monochromator-based illumination system under the control of TillVision 4.0 software. Fura-2 was excited at 340/380 nm and emitted fluorescence was captured using a 440 nm long-pass filter at 20X magnification.

For HEK-293 cells (Figures 2A and S2A), Fura-2-loaded cells on coverslips were adhered to a Warner perfusion chamber using vacuum grease and perfused with HBS2. Fura-2 measurement was performed using an inverted epifluorescence Nikon microscope equipped with a 40X oil immersion objective lens. Cells were alternately excited at 340 and 380 nm, and emission was monitored above 505 nm. Images were captured every second with an exposure of 15 ms and 4 × 4 binning using a digital camera (Sensicam QE). Image acquisition was performed using TILLvisION 4.0.^55^

For rat brain microvascular endothelial cells, DAF-FM-loaded cells on coverslips were mounted in an open bath chamber (Warner Instruments, Hamden, CT) on the stage of an inverted Nikon Eclipse TiE microscope (Nikon Inc., Melville, NY). The microscope was equipped with a 40× oil immersion objective lens, Photometrics CoolSnap HQ2 CCD camera (Photometrics, Tucson, AZ), and a Perfect Focus System. During the experiments, the Perfect Focus System was activated. DAF-FM fluorescence (excitation/emission – 480 nm/540 nm) was acquired at a frequency 0.1 Hz using NIS-Elements AR 3.1 software.

#### Subcellular imaging

Elementary cytosolic Ca^2+^ signals HEK-293 cells were measured using Cal-520 and TIRF microscopy. Prior to imaging, the cells were washed three times with HBS2. The cells were subsequently incubated with Cal520-AM (5 μM; AAT Bioquest) and ci-IP_3_/PM (0.5 μM, Tocris) in HBS2 supplemented with 1% BSA in dark at room temperature. After 1-h incubation, the cells were washed three times with HBS2 and incubated in HBS2 containing EGTA-AM (5 μM, Invitrogen). After 45 min incubation, the media was replaced with fresh HBS2 and incubated for additional 30 min at room temperature to allow for de-esterification of loaded reagents.^56^

Following loading, the coverslip was mounted on a chamber and imaged using an Olympus IX83 inverted total internal reflection fluorescence microscopy (TIRFM) equipped with an oil-immersion PLAPO OTIRFM 60x objective lens/1.45 numerical aperture. The cells were illuminated using a 488 nm laser to excite Cal-520 and the emitted fluorescence was collected through a band-pass filter by a Hamamatsu ORCA-Fusion CMOS camera. The angle of the excitation beam was adjusted to achieve TIRF with a penetration depth of ~140 nm. Images were captured from a field of view by directly streaming into RAM. TIRF images were captured using 2 X 2-pixel binning (216 nm/pixel) from equal field of views for both HEK-293 and HEK-3KO cells at a rate of ~50 frames per second. Agonists were applied directly to the imaging chamber and ciIP_3_ was uncaged by delivering a UV flash from a 405 nm laser uniformly to uncage ci-IP_3_ for 1 s when indicated.

After visualizing images with the cellSens [Ver.2.3] life science imaging software (Olympus), images were exported as vsi files as described in.^57^ The vsi files were converted to TIFF files using ImageJ 1.53f51 and further processed using FLIKA (Ver 1), a Python programming-based tool for image processing.^50^ From each recording, 100 frames (~2 s) before agonist stimulation were averaged to obtain a ratio image stack (F/F0) and standard deviation for each pixel for recording up to 30 s following photolysis. The image stack was Gaussian-filtered, and pixel that exceeded a critical value (0.8 for our analysis) were located. The ‘Detect-puffs’ plug-in was utilized to detect the number of clusters, number of events, amplitudes and durations of localized Ca^2+^ signals from equal areas across different conditions from individual cells. All the puffs identified automatically by the algorithm were manually confirmed before further analysis.^46,58^

#### Cell population measurements

Cytosolic Ca^2+^ in populations of HeLa and U2OS cells was measured using Fura-2 and a fluorescence plate reader (Clariostar, BMG Labtech) under the control of Mars 3.42 R3 software. Cells were incubated with Fura-2-AM (2.5 μM) and 0.005% (v/v) pluronic acid (from Invitrogen) for 1 h in HBS1. A single measurement comprised 16 flashes at 335 nm and 380 nm (each at 8 nm bandpass) while recording fluorescence at 520 nm (90 nm bandpass). Measurements were repeated on an individual well at 3 s intervals. Defined volumes of TPC2-A1-N, TPC2-A1-P and histamine were added simultaneously through two independent injector needles to achieve the indicated final concentrations. Background fluorescence was measured from wells containing cells that were incubated with HBS1 without Fura-2.

### QUANTIFICATION AND STATISTICAL ANALYSIS

#### Statistics

Parametric tests were performed using Unpaired t test, two-tail, One-way ANOVA followed by Dunnett’s test or two-way ANOVA followed by Bonferroni’s test. Non-parametric tests were performed using Kruskal-Wallis analysis followed by Dunn’s test. All data were analyzed using Prism 9 (GraphPad Software). Values for n and the exact statistical test used are detailed in the figure legends. *p < 0.05 **p < 0.01 ***p < 0.001 ****p < 0.0001, n.s. not significant.

## Supplementary Material

1

2

3

4

5

6

7

## Figures and Tables

**Table:** KEY RESOURCES TABLE

REAGENT or RESOURCE	SOURCE	IDENTIFIER

**Chemicals, peptides, and recombinant proteins**		

Fura-2-AM	Biotium (for HeLa and U2OS cells)	Cat# 50033-1
Fura-2-AM	Invitrogen (for HEK cells)	Cat# F1221
Cal520-AM	AAT Bioquest	Cat# 21130
Fluorescein dextran	Invitrogen	Cat# D1821
DAF-FM Diacetate	Invitrogen	Cat# D23844
EGTA-AM	ChemCruz (for HeLa cells)	Cat# sc-495800
EGTA-AM	Invitrogen (for HEK cells)	Cat# E1219
BAPTA-AM	Cayman	Cat# 15551
ci-IP_3_/PM	Tocris	Cat# 6210
Pluronic acid	Invitrogen	Cat# P3000MP
TPC2-A1-N	Franz lab^17^ (for HeLa, U2OS and HEK cells)	N/A
TPC2-A1-N	Sigma (for rat brain microvascular endothelial cells)	Cat# SML3562
TPC2-A1-P	Franz lab^17^	N/A
Ionomycin	Cayman	Cat# 11932
Thapsigargin	ChemCruz	Cat# sc-24017
Tetrandrine	ChemCruz	Cat# sc-201492A
Histamine	Sigma	Cat# H7125

**Critical commercial assays**		

Round 13mm coverslips	Academy	Cat# 400-12-21
Round 25mm coverslips	Warner Instruments	Cat# 64-0715
Round 15mm coverslips	Warner Instruments	Cat# 64-0703
96 well plate, black/clear bottom	Corning	Cat# CC691
Lipofectamine^™^ 2000	Invitrogen	Cat# 11668-027
Hanks’ Balanced Salt Solution (HBSS)	Corning	Cat# 21-923-CV

**Experimental models: Cell lines**		

HEK-293 3KO cells engineered using CRISPR-Cas9 technology to lack all three IP3 receptor isoforms.	Yule lab^36^	N/A
Rat brain microvascular endothelial cells	Cell Applications, Inc	Cat# 840-05a

**Recombinant DNA**		

TPC2-GFP	Patel lab^15^	N/A
LAMP1-GFP	Addgene^48^	Plasmid #3483
TPC2^L265P^-GFP	Patel lab^37^	N/A
TRPML1-GFP	Dr S Muallem, NIH, MD, USA^49^	N/A

**Software and algorithms**		

TillVision 4.0	Till Photonics	N/A
MetaFluor 7.10.3.279	Molecular Devices	
FLIKA	https://flika-org.github.io/ ^50^	N/A
CellSens Dimensions 2.3 (Build 189987)	Olympus	N/A
Mars 3.42 R3	BMG Labtech	N/A
NIS-Elements AR 3.1	Nikon Inc.	N/A
Prism9	GraphPad Software	N/A

## Highlights

- Lysosomal TPC2 channels evoke local and global Ca^2+^ signals involving the ER
- ER-localized IP_3_ receptor channels locally couple to TPC2
- TPC2 sensitizes IP_3_ receptors in an agonist-selective manner
- Crosstalk regulates physiological Ca^2+^ signals and Ca^2+^-dependent function

## References

[1] Clapham DE. (2007). Calcium signaling. Cell 131, 1047–1058. 18083096 10.1016/j.cell.2007.11.028

[2] Nelson MT, Cheng H, Rubart M, Santana LF, Bonev AD, Knot HJ, and Lederer WJ. (1995). Relaxation of arterial smooth muscle by calcium sparks. Science 270, 633–637. 7570021 10.1126/science.270.5236.633

[3] Bootman MD, and Bultynck G. (2020). Fundamentals of Cellular Calcium Signaling: A Primer. Cold Spring Harb. Perspect. Biol. 12, a038802. 10.1101/cshperspect.a038802 PMC6942118 31427372

[4] Berridge MJ. (2016). The Inositol Trisphosphate/Calcium Signaling Pathway in Health and Disease. Physiol. Rev. 96, 1261–1296. 27512009 10.1152/physrev.00006.2016

[5] Foskett JK, White C, Cheung KH, and Mak DOD. (2007). Inositol trisphosphate receptor Ca^2+^ release channels. Physiol. Rev. 87, 593–658. 17429043 10.1152/physrev.00035.2006 PMC2901638

[6] Arige V, Terry LE, Wagner LE 2nd, Malik S, Baker MR, Fan G, Joseph SK, Serysheva II, and Yule DI. (2022). Functional determination of calcium-binding sites required for the activation of inositol 1,4,5-trisphosphate receptors. Proc. Natl. Acad. Sci. USA 119, e2209267119. 10.1073/pnas.2209267119 PMC9522344 36122240

[7] Iino M. (1990). Biphasic Ca^2+^ dependence of inositol 1,4,5-trisphosphate-induced Ca^2+^ release in smooth muscle cells of the guinea pig taenia caeci. J. Gen. Physiol. 95, 1103–1122. 2373998 10.1085/jgp.95.6.1103 PMC2216357

[8] Sun XP, Callamaras N, Marchant JS, and Parker I. (1998). A continuum of InsP3-mediated elementary Ca2+ signalling events in Xenopus oocytes. J. Physiol. 509, 67–80. 9547382 10.1111/j.1469-7793.1998.067bo.x PMC2230949

[9] Marchant JS, and Parker I. (2001). Role of elementary Ca^2+^ puffs in generating repetitive Ca^2+^ oscillations. EMBO J. 20, 65–76. 11226156 10.1093/emboj/20.1.65 PMC140189

[10] Boittin FX, Macrez N, Halet G, and Mironneau J. (1999). Norepinephrine-induced Ca(2+) waves depend on InsP(3) and ryanodine receptor activation in vascular myocytes. Am. J. Physiol. 277, C139–C151. 10409117 10.1152/ajpcell.1999.277.1.C139

[11] Phillips B, Clark J, Martineau É Rungta RL, and Orai RR. (2023). IP(3)R channels cooperatively regulate calcium signaling in brain midcapillary pericytes. Commun. Biol. 6, 493. 37149720 10.1038/s42003-023-04858-3 PMC10164186

[12] Yamasaki M, Thomas JM, Churchill GC, Garnham C, Lewis AM, Cancela JM, Patel S, and Galione A. (2005). Role of NAADP and cADPR in the induction and maintenance of agonist-evoked Ca^2+^ Spiking in mouse pancreatic acinar cells. Curr. Biol. 15, 874–878. 15886108 10.1016/j.cub.2005.04.033

[13] Galione A, Morgan AJ, Arredouani A, Davis LC, Rietdorf K, Ruas M, and Parrington J. (2010). NAADP as an intracellular messenger regulating lysosomal calcium-release channels. Biochem. Soc. Trans. 38, 1424–1431. 21118101 10.1042/BST0381424

[14] Calcraft PJ, Ruas M, Pan Z, Cheng X, Arredouani A, Hao X, Tang J, Rietdorf K, Teboul L, Chuang KT, (2009). NAADP mobilizes calcium from acidic organelles through two-pore channels. Nature 459, 596–600. 19387438 10.1038/nature08030 PMC2761823

[15] Brailoiu E, Churamani D, Cai X, Schrlau MG, Brailoiu GC, Gao X, Hooper R, Boulware MJ, Dun NJ, Marchant JS, and Patel S. (2009). Essential requirement for two-pore channel 1 in NAADP-mediated calcium signaling. J. Cell Biol. 186, 201–209. 19620632 10.1083/jcb.200904073 PMC2717647

[16] Patel S. (2015). Function and dysfunction of two-pore channels. Sci. Signal. 8, re7. 10.1126/scisignal.aab3314 26152696

[17] Gerndt S, Chen CC, Chao YK, Yuan Y, Burgstaller S, Scotto Rosato A, Krogsaeter E, Urban N, Jacob K, Nguyen ONP, (2020). Agonist-mediated switching of ion selectivity in TPC2 differentially promotes lysosomal function. Elife 9, e54712. 10.7554/eLife.54712 PMC7108868 32167471

[18] Yuan Y, Jaślan D, Rahman T, Bolsover SR, Arige V, Wagner LE 2nd, Abrahamian C, Tang R, Keller M, Hartmann J, (2022). Segregated cation flux by TPC2 biases Ca(2+) signaling through lysosomes. Nat. Commun. 13, 4481. 35918320 10.1038/s41467-022-31959-0 PMC9346130

[19] Patel S, Yuan Y, Gunaratne GS, Rahman T, and Marchant JS. (2022). Activation of endo-lysosomal two-pore channels by NAADP and PI(3,5)P(2). Five things to know. Cell Calcium 103, 102543. 10.1016/j.ceca.2022.102543 PMC9552313 35123238

[20] Gunaratne GS, Brailoiu E, He S, Unterwald EM, Patel S, Slama JT, Walseth TF, and Marchant JS. (2021). Essential requirement for JPT2 in NAADP-evoked Ca(2+) signaling. Sci. Signal. 14, eabd5605. 10.1126/scisignal.abd5605 PMC8315109 33758061

[21] Zhang J, Guan X, Shah K, and Yan J. (2021). Lsm12 is an NAADP receptor and a two-pore channel regulatory protein required for calcium mobilization from acidic organelles. Nat. Commun. 12, 4739. 34362892 10.1038/s41467-021-24735-z PMC8346516

[22] Marchant JS, Gunaratne GS, Cai X, Slama JT, and Patel S. (2022). NAADP binding proteins find their identity. Trends Biochem. Sci. 47, 235–249. 34810081 10.1016/j.tibs.2021.10.008 PMC8840967

[23] Vassileva K, Marsh M, and Patel S. (2020). Two-pore channels as master regulators of membrane trafficking and endocytic well-being. Curr. Opin. Physiol. 17, 163–168. 32838099 10.1016/j.cophys.2020.08.002 PMC7426208

[24] Cancela JM, Churchill GC, and Galione A. (1999). Coordination of agonist-induced Ca^2+^-signalling patterns by NAADP in pancreatic acinar cells. Nature 398, 74–76. 10078532 10.1038/18032

[25] Patel S, Churchill GC, and Galione A. (2001). Coordination of Ca^2+^ signalling by NAADP. Trends Biochem. Sci. 26, 482–489. 11504624 10.1016/s0968-0004(01)01896-5

[26] Galione A. (2015). A primer of NAADP-mediated Ca signalling: From sea urchin eggs to mammalian cells. Cell Calcium 58, 27–47. 25449298 10.1016/j.ceca.2014.09.010

[27] Heister PM, Powell T, and Galione A. (2021). Glucose and NAADP trigger elementary intracellular β-cell Ca(2+) signals. Sci. Rep. 11, 10714. 10.1038/s41598-021-88906-0 PMC8140081 34021189

[28] Brailoiu GC, Gurzu B, Gao X, Parkesh R, Aley PK, Trifa DI, Galione A, Dun NJ, Madesh M, Patel S, (2010). Acidic NAADP-sensitive calcium stores in the endothelium: agonist-specific recruitment and role in regulating blood pressure. J. Biol. Chem. 285, 37133–37137. 10.1074/jbc.C110.169763 PMC2988319 20876534

[29] Esposito B, Gambara G, Lewis AM, Palombi F, D’Alessio A, Taylor LX, Genazzani AA, Ziparo E, Galione A, Churchill GC, and Filippini A. (2011). NAADP links histamine H1 receptors to secretion of von Willebrand factor in human endothelial cells. Blood 117, 4968–4977. 21364192 10.1182/blood-2010-02-266338

[30] Berra-Romani R, Faris P, Pellavio G, Orgiu M, Negri S, Forcaia G, Var-Gaz-Guadarrama V, Garcia-Carrasco M, Botta L, Sancini G, (2020). Histamine induces intracellular Ca(2+) oscillations and nitric oxide release in endothelial cells from brain microvascular circulation. J. Cell. Physiol. 235, 1515–1530. 31310018 10.1002/jcp.29071

[31] Negri S, Faris P, and Moccia F. (2021). Endolysosomal Ca(2+) signaling in cardiovascular health and disease. Int. Rev. Cell Mol. Biol. 363, 203–269. 34392930 10.1016/bs.ircmb.2021.03.001

[32] Stern MD. (1992). Buffering of calcium in the vicinity of a channel pore. Cell Calcium 13, 183–192. 1315621 10.1016/0143-4160(92)90046-u

[33] Sakurai Y, Kolokoltsov AA, Chen CC, Tidwell MW, Bauta WE, Klugbauer N, Grimm C, Wahl-Schott C, Biel M, and Davey RA. (2015). Two-pore channels control Ebola virus host cell entry and are drug targets for disease treatment. Science 347, 995–998. 25722412 10.1126/science.1258758 PMC4550587

[34] Patel S, Joseph SK, and Thomas AP. (1999). Molecular properties of inositol 1,4,5-trisphosphate receptors. Cell Calcium 25, 247–264. 10378086 10.1054/ceca.1999.0021

[35] Baker MR, Fan G, Arige V, Yule DI, and Serysheva II. (2023). Understanding IP(3)R channels: From structural underpinnings to ligand-dependent conformational landscape. Cell Calcium 114, 102770. 10.1016/j.ceca.2023.102770 PMC10529787 37393815

[36] Alzayady KJ, Wang L, Chandrasekhar R, Wagner LE 2nd, Van Petegem F, and Yule DI. (2016). Defining the stoichiometry of inositol 1,4,5-trisphosphate binding required to initiate Ca2+ release. Sci. Signal. 9, ra35. 10.1126/scisignal.aad6281 PMC4850551 27048566

[37] Brailoiu E, Rahman T, Churamani D, Prole DL, Brailoiu GC, Hooper R, Taylor CW, and Patel S. (2010). An NAADP-gated two-pore channel targeted to the plasma membrane uncouples triggering from amplifying Ca2+ signals. J. Biol. Chem. 285, 38511–38516. 20880839 10.1074/jbc.M110.162073 PMC2992283

[38] Saito R, Mu Q, Yuan Y, Rubio-Alarcó n M, Eznarriaga M, Zhao P, Gunaratne G, Kumar S, Keller M, Bracher F, (2023). Convergent activation of Ca(2+) permeability in two-pore channel 2 through distinct molecular routes. Sci. Signal. 16, eadg0661. 10.1126/scisignal.adg0661 PMC10639088 37607219

[39] Patel S. (2004). NAADP-induced Ca^2+^ release - a new signaling pathway. Biol. Cell 96, 19–28. 15093124 10.1016/j.biolcel.2003.12.001

[40] Guse AH, and Lee HC. (2008). NAADP: a universal Ca^2+^ trigger. Sci. Signal. 1, re10. 10.1126/scisignal.144re10 18984909

[41] Cancela JM, Van Coppenolle F, Galione A, Tepikin AV, and Petersen OH. (2002). Transformation of local Ca^2+^ spikes to global Ca^2+^ transients: the combinatorial roles of multiple Ca^2+^ releasing messengers. EMBO J. 21, 909–919. 11867519 10.1093/emboj/21.5.909 PMC125894

[42] Yamasaki M, Masgrau R, Morgan AJ, Churchill GC, Patel S, Ashcroft SJH, and Galione A. (2004). Organelle selection determines agonist-specific Ca^2+^ signals in pancreatic acinar and beta cells. JBC 279, 7234–7240. 10.1074/jbc.M311088200 14660554

[43] Pandey V, Chuang CC, Lewis AM, Aley PK, Brailoiu E, Dun NJ, Churchill GC, and Patel S. (2009). Recruitment of NAADP-sensitive acidic Ca2+ stores by glutamate. Biochem. J. 422, 503–512. 19548879 10.1042/BJ20090194

[44] Bers DM. (2002). Cardiac excitation-contraction coupling. Nature 415, 198–205. 11805843 10.1038/415198a

[45] She J, Zeng W, Guo J, Chen Q, Bai XC, and Jiang Y. (2019). Structural mechanisms of phospholipid activation of the human TPC2 channel. Elife 8, e45222. 10.7554/eLife.45222 PMC6424560 30860481

[46] Lock JT, Alzayady KJ, Yule DI, and Parker I. (2018). All three IP(3) receptor isoforms generate Ca(2+) puffs that display similar characteristics. Sci. Signal. 11, eaau0344. 10.1126/scisignal.aau0344 PMC6402561 30563861

[47] Thakore P, Pritchard HAT, Griffin CS, Yamasaki E, Drumm BT, Lane C, Sanders KM, Feng Earley Y, and Earley S. (2020). TRPML1 channels initiate Ca(2+) sparks in vascular smooth muscle cells. Sci. Signal. 13, eaba1015. 10.1126/scisignal.aba1015 PMC7397860 32576680

[48] Falcó n-Pé rez JM, Nazarian R, Sabatti C, and Dell’Angelica EC. (2005). Distribution and dynamics of Lamp1-containing endocytic organelles in fibroblasts deficient in BLOC-3. J. Cell Sci. 118, 5243–5255. 16249233 10.1242/jcs.02633

[49] Yamaguchi S, Jha A, Li Q, Soyombo AA, Dickinson GD, Churamani D, Brailoiu E, Patel S, and Muallem S. (2011). Transient receptor potential mucolipin 1 (TRPML1) and two-pore channels are functionally independent organellar ion channels. J. Biol. Chem. 286, 22934–22942. 10.1074/jbc.M110.210930 PMC3123061 21540176

[50] Ellefsen KL, Settle B, Parker I, and Smith IF. (2014). An algorithm for automated detection, localization and measurement of local calcium signals from camera-based imaging. Cell Calcium 56, 147–156. 25047761 10.1016/j.ceca.2014.06.003 PMC4162823

[51] Leo LM, Familusi B, Hoang M, Smith R, Lindenau K, Sporici KT, Brailoiu E, Abood ME, and Brailoiu GC. (2019). GPR55-mediated effects on brain microvascular endothelial cells and the blood-brain barrier. Neuroscience 414, 88–98. 31279825 10.1016/j.neuroscience.2019.06.039 PMC6684354

[52] Lindenau KL, Barr JL, Higgins CR, Sporici KT, Brailoiu E, and Brailoiu GC. (2022). Blood-Brain Barrier Disruption Mediated by FFA1 Receptor-Evidence Using Miniscope. Int. J. Mol. Sci. 23, 2258. 35216375 10.3390/ijms23042258 PMC8875452

[53] Altmann JB, Yan G, Meeks JF, Abood ME, Brailoiu E, and Brailoiu GC. (2015). G protein-coupled estrogen receptor-mediated effects on cytosolic calcium and nanomechanics in brain microvascular endothelial cells. J. Neurochem. 133, 629–639. 25703621 10.1111/jnc.13066 PMC4562690

[54] Brailoiu E, Barlow CL, Ramirez SH, Abood ME, and Brailoiu GC. (2018). Effects of Platelet-Activating Factor on Brain Microvascular Endothelial Cells. Neuroscience 377, 105–113. 29522856 10.1016/j.neuroscience.2018.02.039 PMC5882569

[55] Terry LE, Arige V, Neumann J, Wahl AM, Knebel TR, Chaffer JW, Malik S, Liston A, Humblet-Baron S, Bultynck G, and Yule DI. (2022). Missense mutations in inositol 1,4,5-trisphosphate receptor type 3 result in leaky Ca(2+) channels and activation of store-operated Ca(2+) entry. iScience 25, 105523. 10.1016/j.isci.2022.105523 PMC9700043 36444295

[56] Lock JT, Ellefsen KL, Settle B, Parker I, and Smith IF. (2015). Imaging local Ca2+ signals in cultured mammalian cells. J. Vis. Exp. 97. 52516. 10.3791/52516 PMC4401178 25867132

[57] Arige V, Emrich SM, Yoast RE, Trebak M, and Yule DI. (2021). A protocol for detecting elemental calcium signals (Ca(2+) puffs) in mammalian cells using total internal reflection fluorescence microscopy. STAR Protoc. 2, 100618. 10.1016/j.xpro.2021.100618 PMC8225975 34195673

[58] Arige V, Terry LE, Malik S, Knebel TR, Wagner Ii LE, and Yule DI. (2021). CREB regulates the expression of type 1 inositol 1,4,5-trisphosphate receptors. J. Cell Sci. 134, jcs258875. 10.1242/jcs.258875 PMC8601716 34533188

