# Supplementary material for: Two-pore channel-2 and inositol trisphosphate receptors coordinate Ca2+ signals between lysosomes and the endoplasmic reticulum
Source: Cell Rep. Author manuscript; Available in PMC 2024 Mar 12. (PMC10931537; doi:10.1016/j.celrep.2023.113628)
Supplement: 1 [file NIHMS1961507-supplement-1.pdf]

Cell Reports, Volume *43*

Supplemental information

Two-pore channel-2 and inositol trisphosphate receptors coordinate Ca^{2+} signals between lysosomes and the endoplasmic reticulum

Yu Yuan, Vikas Arige, Ryo Saito, Qianru Mu, Gabriela C. Brailoiu, Gustavo J.S. Pereira, Stephen R. Bolsover, Marco Keller, Franz Bracher, Christian Grimm, Eugen Brailoiu, Jonathan S. Marchant, David I. Yule, and Sandip Patel

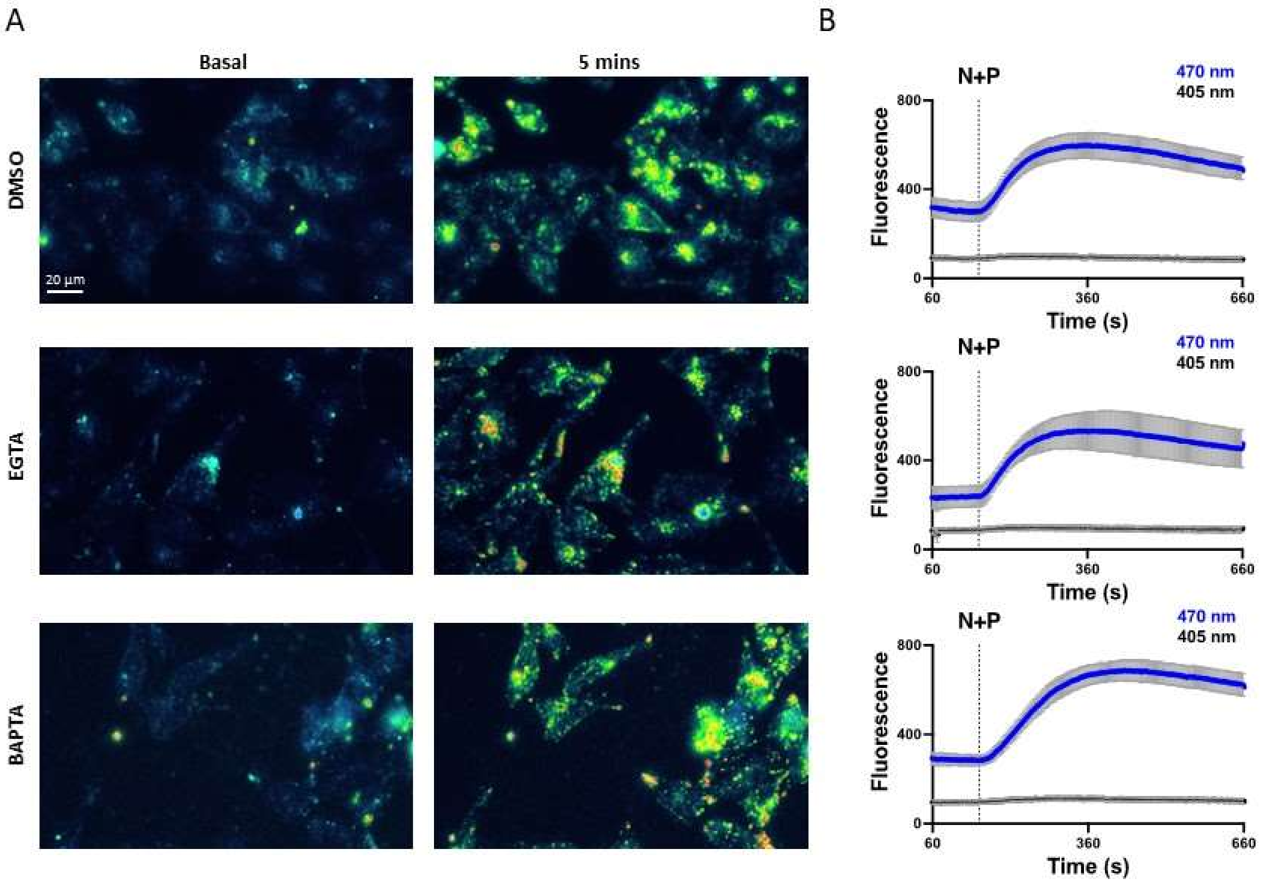

Figure S1. Validation of lysosomal pH measurements, related to Figure 1.

A, Exemplar pseudo-coloured images depicting the fluorescence ratio of endocytosed fluorescein dextran before and after stimulation with a combination of TPC2-A1-N (30 µM) and TPC2-A1-P (60 µM). Cells were pre-treated with the indicated AM ester of EGTA (50 µM) or BAPTA-AM (10 µM).

B, Exemplar time-courses showing corresponding changes in fluorescence upon excitation at pH sensitive (470 nm) and insensitive (405 nm) wavelengths. Each trace is the fluorescence ratio response of all cells imaged from a typical field of view (mean ± s.e.m. of 15-30 technical replicates). External Ca^{2+} was removed prior to stimulation.

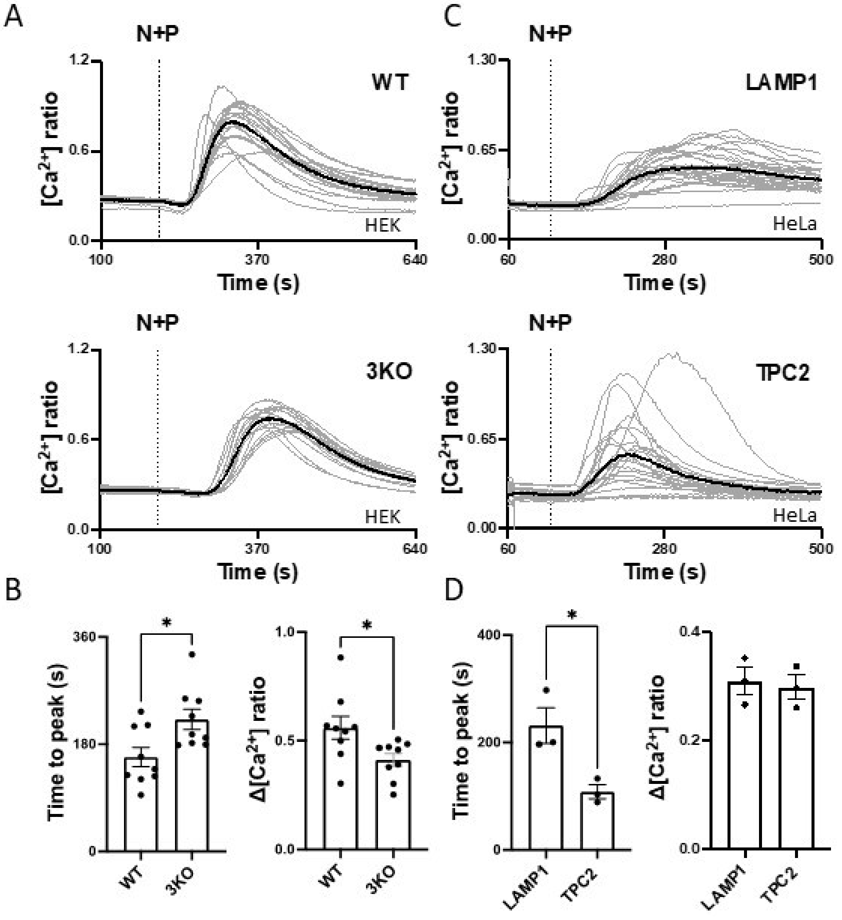

Figure S2. ER and lysosomal channel levels set the timing of TPC2-evoked Ca^{2+} signals, related to Figure 2.

A, Effect of a combination of TPC2-A1-N (15 μM) and TPC2-A1-P (20 μM) (N+P) on cytosolic Ca^{2+} in individual wild-type (WT) and IP_3 receptor knockout (3KO) HEK-293 cells loaded with Fura-2. Each trace is the fluorescence ratio response of a single cell imaged from a typical field of view. The thicker trace is the average of the population. External Ca^{2+} was removed prior to stimulation.

B, Pooled data (mean ± s.e.m. from 9 biological replicates) quantifying the maximal change and the time to peak of the cytosolic Ca^{2+} signals in response to the TPC2-A1-N and TPC2-A1-P combination in HEK-293 cells. Each point represents the mean response of all cells from an independent experiment. *$p<0.05$ (Unpaired t-test, two-tailed)

C, Effect of combination of TPC2-A1-N (30 μM) and TPC2-A1-P (60 μM) (N+P) on cytosolic Ca^{2+} in individual HeLa cells transiently transfected with TPC2-GFP and LAMP1-GFP. Each trace is the fluorescence ratio response of a single cell imaged from a typical field of view. The thicker trace is the average of the population. External Ca^{2+} was removed prior to stimulation.

D, Pooled data (mean ± s.e.m. from 3 biological replicates) quantifying the maximal change and the time to peak of the cytosolic Ca^{2+} signals in response to the TPC2-A1-N and TPC2-A1-P combination in HeLa cells. Each point represents the mean response of all cells from an independent experiment. *$p<0.05$ (Unpaired t-test, two-tailed)

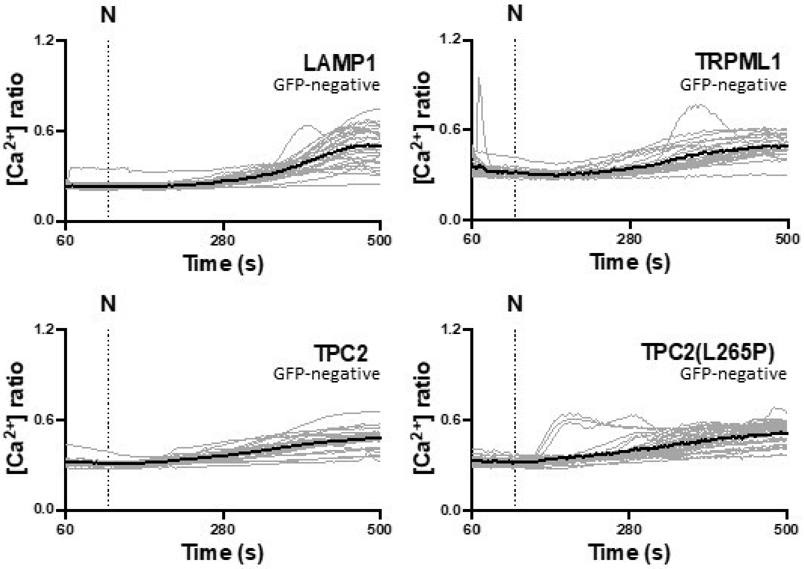

Figure S3. Validation of Ca^{2+} measurements upon transient transfection, related to Figure 2.

Effect of TPC2-A1-N (30 μM) on cytosolic Ca^{2+} in individual GFP-negative cells from a population of HeLa cells transiently transfected with LAMP1-GFP, TPC2-GFP, TRPML1-GFP or $TPC2^{L265P}$-GFP and loaded with Fura-2. Each trace is the fluorescence ratio response of a single cell imaged from a typical field of view. The thicker trace is the average of the population. External Ca^{2+} was removed prior to stimulation.

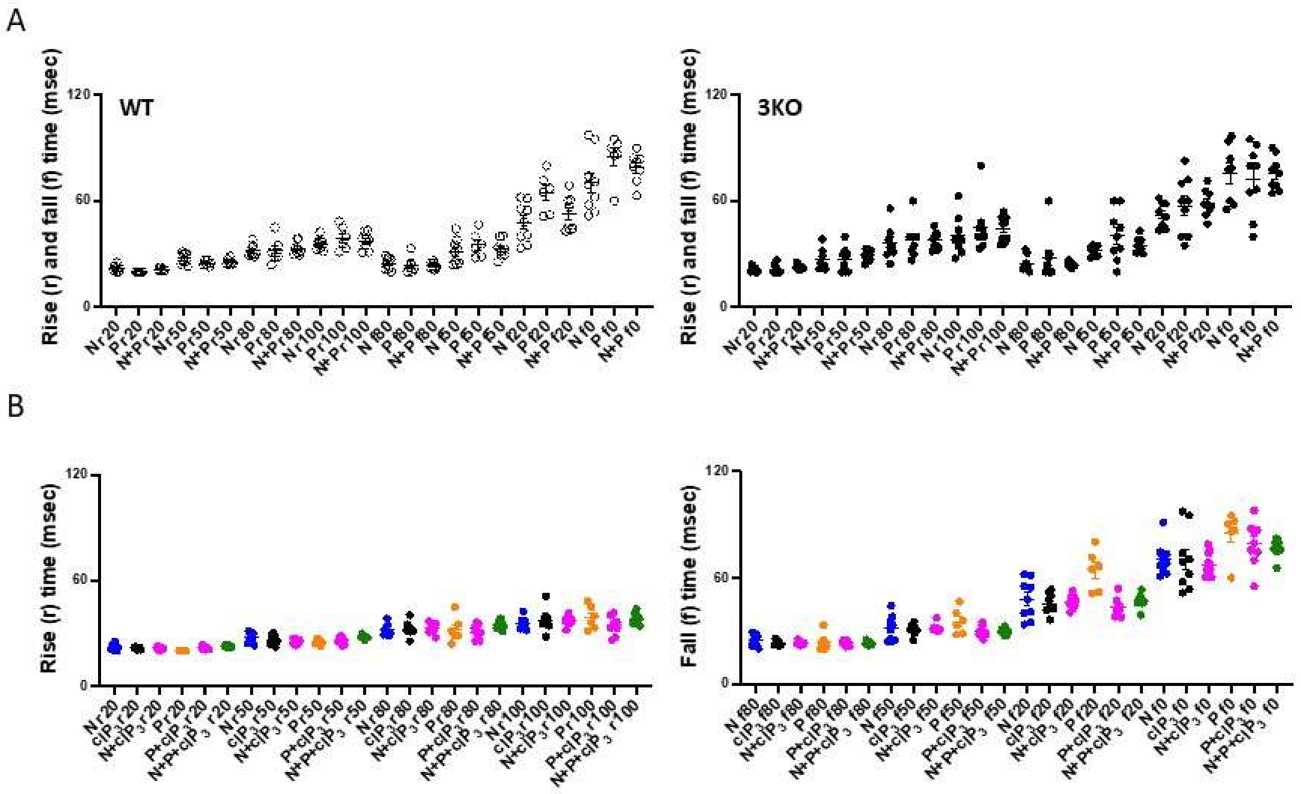

Figure S4. Kinetics of elementary Ca^{2+} signals, related to Figures 3 and 4.

A, Pooled data (mean ± s.e.m. from 6-9 biological replicates) showing the mean rise (r) and fall (f) times of tuffs to the indicated stimulus recorded from individual wild-type (WT) and IP_3 receptor knockout (3KO) HEK cells loaded with Cal-520. Data were calculated to the indicated normalized intensity level for an individual event.

B, Pooled data (mean ± s.e.m. from 6-10 biological replicates) showing the mean rise (r) and fall (f) times of tuffs or puffs to the indicated stimulus recorded from individual HEK cells loaded with Cal-520. Data were calculated to the indicated normalized intensity level for an individual event.

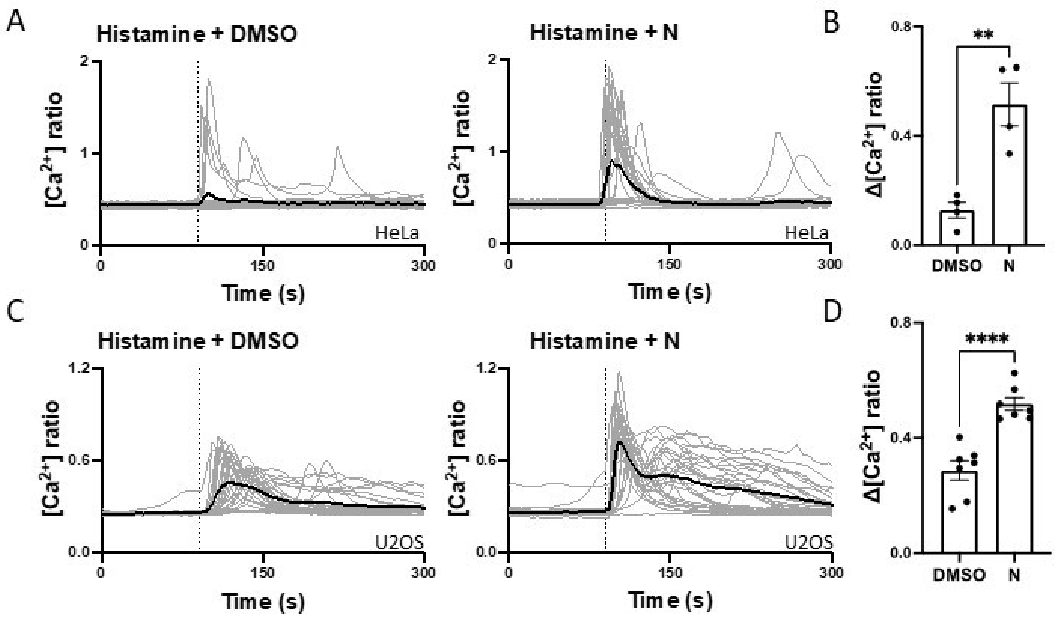

Figure S5. Validation of automated Ca^{2+} measurements in cell populations, related to Figure 5.

A, Effect of TPC2-A1-N (30 µM) on Ca^{2+} signals induced by histamine (2.6 µM) in individual HeLa cells loaded with Fura-2. Each trace is the fluorescence ratio response of a single cell imaged from a typical field of view. The thicker traces are the average of the population.

B, Pooled data (mean ± s.e.m. from 4 biological replicates) quantifying the effect of TPC2-A1-N on the peak change in cytosolic Ca^{2+} in response to histamine in HeLa cells. Each point represents the mean response of all cells from an independent experiment. **$p<0.01$ (Unpaired t-test, two-tailed).

C, Effect of TPC2-A1-N (10 µM) on Ca^{2+} signals induced by histamine (2.6 µM) in individual U2OS cells loaded with Fura-2. Each trace is the fluorescence ratio response of a single cell imaged from a typical field of view. The thicker traces are the average of the population.

D, Pooled data (mean ± s.e.m. from 7 biological replicates) quantifying the effect of TPC2-A1-N on the peak change in cytosolic Ca^{2+} in response to histamine in U2OS cells. Each point represents the mean response of all cells from an independent experiment. ****$p<0.0001$ (Unpaired t-test, two-tailed).
